# The value of virtual glaucoma clinics: a review

**DOI:** 10.1038/s41433-024-03056-7

**Published:** 2024-04-08

**Authors:** Rachel Mercer, Pouya Alaghband

**Affiliations:** Ophthalmology Department, York Hospital, Wigginton Road, York, YO318HE UK

**Keywords:** Health services, Diseases

## Abstract

Virtual clinics are being utilised to tackle the growing demand for glaucoma healthcare. We conducted a literature search on 28 February 2023 using MEDLINE (PubMed), EMBASE and Web of Science databases. We searched for studies on virtual glaucoma clinics, published in the English language between 2000 and 2023. Studies suggest that virtual glaucoma clinics are a safe and effective alternative to traditional face-to-face clinics for patients with stable and early-to-moderate glaucoma. Patient satisfaction is high across all clinics surveyed. Satisfaction appears to be linked to good communication, trust and improved waiting times. The majority of healthcare professionals are also content with virtual glaucoma clinics. There are no dedicated cost-benefit analyses for virtual glaucoma clinics in the UK. However, virtual clinics in other specialties have reported significant cost savings.

## Abstract (zh)

虚拟诊所正用于解决青光眼医疗保健日益增长的需求。我们于2023年2月28日使用MEDLINE (PubMed) 、EMBASE和Web of Science数据库进行了文献检索, 共搜集2000年至2023年间发表的英文研究。研究表明, 对于稳定型和早期至中度青光眼的患者来说, 虚拟青光眼诊所是传统面对面诊所的安全有效的替代方案。所有接受调查的患者满意度都很高。且满意度似乎与良好的沟通、信任和缩短的候诊时间有关。大多数医疗保健专业人员也对虚拟青光眼诊所感到满意。虽然没有专门的青光眼成本效益分析, 但其他虚拟诊所已经报告了显著的成本节约。

## Introduction

The prevalence of glaucoma increases with age [1]. With an aging population, the burden of disease on healthcare services will soar globally [2]. Projections suggest a 44% increase in glaucoma cases; an 18% rise in glaucoma suspects and a 16% increase in ocular hypertension cases by 2035 [3]. Our ability to detect glaucoma in the primary care setting is also improving due to advances in diagnostic modalities [4]. In order to cope with growing numbers in an already stretched Hospital Eye Service, many ophthalmology departments have utilised virtual glaucoma clinics (VGCs) in the United Kingdom [5].

Whilst the first VGC in the UK was set up in Sheffield in 1994, the COVID-19 pandemic has accelerated the virtual revolution [6]. Virtual clinics allow patients to be managed without the need for face-to-face (FTF) consultation [7]. Glaucoma lends itself to the virtual model as functional and structural assessments can be captured by allied healthcare professionals (such as ophthalmic technicians) [8]. Theoretically, the virtual model should improve clinic capacity, reduce healthcare workforce and streamline the patient journey.

This review intends to assess the value of the VGCs. We aim to summarise studies that have evaluated the following questions:

1. Are VGCs as effective as traditional clinics?
2. Are VGCs as safe as traditional clinics?
3. Are VGCs cost effective?
4. What are the opinions of healthcare professionals using VGCs?
5. What are the patients’ views on VGCs?
6. What type of glaucoma is suitable for VGCs?
7. What does the future hold?

## Methods

We conducted a literature search on 28 February 2023 using MEDLINE (PubMed), EMBASE and Web of Science databases. We searched for studies published in the English language between 2000 and 2023. Key search terms included a combination of the following: “virtual”, “telemedicine”, “teleconsultation”, “clinic”, “glaucoma”, “ocular hypertension”, “outpatient”, “telecare” and “tele-glaucoma”. Abstracts were screened before exporting by both authors (RM and PA). Only methodologically sound studies were included. All study designs were accepted.

## Discussion

The findings are summarised in Table 1.

**Table 1:** A summary of the evidence.

Question	Evidence
Are VGCs effective?	All studies show that VGCs are effective at diagnosing and monitoring glaucoma. The efficacy increases if the clinics are conducted by Glaucoma Consultants and if moderate-severe or unstable glaucoma is excluded.
Are VGCs safe?	Misdiagnoses and misclassification rates are low across studies. In optometry led VGCs, glaucoma training and glaucoma specialist review are required to maintain safety. There are mixed reports on the safety of grading iridocorneal angles using AC-OCT. FTF assessments are required for unequivocal cases.
Are VGCs cost effective?	There are no published analyses for VGCs in the UK. However, American data shows they are cost effective there. Additionally, virtual clinics in other specialities in the UK are more cost effective than FTF clinics.
What do Healthcare Professionals think of VGCs?	Most clinicians report high levels of satisfaction with VGCs. The main challenge to implementing VGCs is understaffing.
What do patients think of VGCs?	Patient satisfaction was high across studies. Surveys suggest that patients are happy with the communication. There are high levels of trust in the VGC. Patient waiting times are significantly less in the VGC compared to FTF clinics.
What type of glaucoma is suitable for VGCs?	Low-risk glaucoma. These patients include those with OHT, glaucoma suspects, early and/ or stable glaucoma and pseudophakic angle closure patients.
What does the future hold?	Mobile devices that measure glaucoma parameters may be the future. This allows the patient to send information from home. AI can help with reviewing patient data.

*VGCs* virtual glaucoma clinics, *AC-OCT* anterior chamber optical coherence tomography, *FTF* face-to-face, *OHT* ocular hypertension, *AI* artificial intelligence.

### Are virtual glaucoma clinics effective?

Most studies addressing the effectiveness of VGCs compare the diagnosis or grading of glaucoma in VGCs with FTF clinics. Cohen’s or Fleiss’ kappa coefficient is frequently used to quantify inter- and intra-observer agreement. All studies showed some agreement between VGCs and FTF clinics and no studies showed disagreement. The level of agreement varied from fair to good (kappa 0.21–0.72) [9–15]. A study looking at accuracy of VGCs in diagnosing glaucoma showed that there was a sensitivity of 86.2% and a specificity of 82.1% [16]. These studies ultimately demonstrated that VGCs appear to be effective.

Agreement was more likely when both FTF clinics and VGCs were conducted by a specialist clinician or a consultant as opposed to an optometrist [10, 11, 13]. Agreement was also higher if patients with moderate-to-severe or unstable glaucoma were excluded [9, 14, 17]. This is likely due to the challenges in assessing these patients and the variability in the management of more severe cases between health care professionals.

Other issues of agreement were related to disc imaging interpretation. There was weaker agreement when it came to large and small discs, peripapillary atrophy and abnormalities of the shape (e.g. tilting) [11, 13]. One study reported only slight agreement between slit lamp examination in FTF clinics compared with virtual assessment for the following optic disc features: peripapillary atrophy, notching and disc haemorrhages [12]. It concludes therefore, that if disc anomalies or abnormalities exist, they should be reviewed as part of a series of assessments, rather than in isolation. This allows for detection of change over time.

### Are virtual glaucoma clinics safe?

Across studies investigating the safety of VGCs, there were no misdiagnoses reported and misclassification (i.e. incorrectly graded glaucoma) rates were low [11, 14, 17]. Specifically, there were no reports of irreversible vision loss caused by a misdiagnosis [18]. One study reported that 0.3–3.8% of assessments were misclassified [14]. The authors also argue that glaucoma is slow to progress in most cases and there are usually periodic re-assessments. This means that in the event of a misclassification, patients should be identified before irreversible damage occurs [13].

One study used big data to compare visual field (VF) progression between those attending FTF clinics and VGCs. A database of VF tests from 18,926 eyes attending FTF clinics across several hospitals was used to create an ‘expected’ range for VF progression measured by mean deviation (MD) [19]. Results showed that patients attending the VGC were well within this expected range [7]. Meaning, the rate of progression is the similar across the two groups. This was echoed in a study looking at around 2000 patients over 31 months in a VGC. They found no clinical deterioration of VGC patients during the period of observation [20].

In optometry led VGCs, safety pivots on two things: glaucoma training and glaucoma specialist review. In a study of 24,257 participants, community optometrists graded patients prior to ophthalmologist review. Fifteen percent of patients that were graded as normal by the optometrists were found to have glaucoma and 6.5% of the patients thought to be stable were actually found to be unstable [10]. The authors do not disclose the level of training of the optometrists. And this is crucial as previous studies demonstrate that specialist optometrists, with glaucoma training, are at the level of glaucoma specialists and work safely within a VGC [21, 22].

A number of virtual referral refinement schemes have been used across the United Kingdom. Patients referred from primary care services attend VGCs in which information is reviewed by a glaucoma specialist. These schemes have proven to reduce the number of false positive referrals to the HES [23–27]. The positive predictive value across the studies is approximately 80%. The highest false negative rate reported in one of these clinics was 20%. Of those, 4% required treatment and 16% required observation [23].

Anterior chamber optical coherence tomography (OCT) can be used to grade the iridocorneal angle. Some studies report diagnostic inaccuracy (i.e. angle grading not possible with the OCT) [23], whilst others report accuracy (i.e. angle grading possible with the OCT) [28–30]. Identifying angle structures may be more difficult for graders because they can be subtle. In the context of a VGC it might be more clinically relevant to label angles as open or closed. A FTF gonioscopy assessment may be required in unequivocal cases of AS-OCT angle assessment [31]. A recent study triaged VGC patients to receive a FTF appointment if there was ≥1 quadrants of iridotrabecular contact on AS-OCT. Of 137 referrals, 66.4% were triaged for a FTF angle assessment. Of these, almost one third were discharged [32]. The Zhongshan Angle Closure Prevention (ZAP) trial demonstrated that prophylactic laser peripheral iridotomies are not required for primary angle closure suspects (PACS) without “PACS PLUS” features. This should mean that the number of patients with narrow angles who need to be evaluated in a hospital setting is much smaller than it once was [33, 34].

Automated gonioscopy may help in the assessment of angles in VGC setting. It is a machine that takes a photograph of the iridocorneal angle. Most studies show agreement using Fleiss’ Kappa coefficient between manual and automated gonioscopy. However, the level of agreement is varied (low to high) [35–37].

The Royal college of Ophthalmologists have published standards for VGCs in the National Health Service (NHS). Patients suitable for VGCs are those with ocular hypertension, suspected open angle glaucoma, early-to moderate primary open angle glaucoma in the worse eye, or bilateral pseudophakia and a primary diagnosis of early-to moderate primary angle closure glaucoma in their worse eye [38]. One pilot study expanded VGC inclusion criteria to patients with various stages of stable glaucoma. They reported no safety concerns. They also assessed *all* new glaucoma cases with good specificity and sensitivity [20]. This may provide additional capacity in glaucoma clinics.

### Are virtual glaucoma clinics cost effective?

There are no published cost-utility analyses for VGCs in the UK. However, in America they have estimated a mean of cost of 89,803 to 123,164 USD to set up a VGC [39]. VGCs are reliant on equipment for data capture and an electronic patient record (EPR). However, in most instances the EPR is already in place. A survey from the European Glaucoma Society (EGS) revealed that only one third of participants had VGCs. Insufficient funding was one of the major setbacks [40]. Whilst there is a large capital cost, using VGCs for screening saves 27,460 USD for each QALY gained and costs 80% less than a FTF clinic equivalent [41].

A VGC in Liverpool estimated that a doctor can review 30–40 patients in a 4-h session [42] Furthermore, in the Singapore National Eye Centre the average time for a doctor to review VGC data was 5.8 min compared with 19.5 min in a FTF clinic. The number of staff required to run and review the VGC was almost half of the conventional clinic [43].

A virtual orthopaedic fracture clinic service was estimated to have a cost saving of approximately £130,000 per annum compared with FTF clinics. Additionally, there was a statistically significant reduction in: new patients seen face-to-face; days to first review; and, non-attendees [44].

### What is the opinion of healthcare professionals using virtual glaucoma clinics?

A 2016 NHS survey revealed that 50% of hospitals used a VGC set up and a further 21% were planning to establish one. The main reason for limited utilisation of VGCs were insufficient staffing (71.4%) and inadequate premises (47.6%) [5]. The EGS survey identified similar reasons for preventing VGC set up. These included a lack of IT systems in place, inadequate staffing, and, insufficient time or funding [40].

The majority of VGCs are used for lower risk patients such as glaucoma suspects and those with ocular hypertension [5]. Two-thirds of EGS survey participants stated that patients with ocular co-morbidities should be followed up in FTF clinics [40]. Most centres implemented VGCs to manage follow-up patients (81%) but some of the respondents were using the virtual clinic model to assess new patients also [5].

All respondents using VGCs were either clinicians or optometrists with a specialist interest or higher qualifications in glaucoma. Ophthalmologists were the main group of reviewers [40]. Surveys suggest that most clinicians have high satisfaction with VGCs [5, 40, 45]. VGCs allow low-risk cases to be reviewed in a timely manner and individuals with progressive disease can be identified effectively. FTF clinics can used for more complex cases who need further examinations or intervention [42].

A wide range of allied healthcare professionals capture data in VGCs such as nurses, ophthalmic technicians and optometrists or orthoptists. Two-thirds of centres have a mix of professionals [40]. A Moorfields Eye Hospital service evaluation study found that a main challenge to implementation of VGCs was understaffing [46]. The staff in the VGCs benefit from training, particularly on glaucoma and related medications. There is high staff satisfaction reported in the VGCs [47].

### What are the patients’ views attending VGCs?

Overall, patient satisfaction was high across the literature [8, 40, 43, 46–52] The main pillars of patient satisfaction were trust, communication and improved waiting times. Average satisfaction scores were 4.3/5 in one survey in all domains [48]. In Plymouth, 98% of patients felt the VGCs were the same or better than traditional FTF clinics [50]. Additionally, a survey conducted in Edinburgh showed 22% of patients strongly agreed and 44% agreed that the VGC was better than FTF clinics [49]. Patients with more advanced disease have a greater appreciation of the importance of regular monitoring [49].

#### Trust

Patients are generally accepting of a virtual service delivery whereby the physical presence of a doctor is excluded from the consultation. Patients should be adequately reassured and informed about the clinic, the status of their condition and their risk of progression to severe sight loss [7]. Most patients demonstrate high levels of trust in the staff performing tests in the VGC [47]. And, patients did not feel the VGCs hinder the doctor-patient relationship [40].

#### Communication

Ninety-seven percent of patients are satisfied with communication received before and after a VGC [46]. In fact, some feel the amount of information is superior to FTF clinics [50]. However, the most recent survey published from Manchester Royal and Bristol Eye Hospitals showed that 10.8% of patients were not happy to have clinic results provided via post. This may be due to visual impairment, disability or language barriers [47]. Therefore, it is crucial to consider individual preferences and requirements before disseminating information.

Healthcare professional’s empathy, competence and communication during the VGC, improves the patient experience [8]. Some patients were concerned about VGC staff abilities to answer questions about their condition. Providing better education may enable them to respond to patient queries [47]. It is important to perform regular audit of the diagnostic, management and communication competencies of all clinical staff [48].

Despite reduced FTF interactions for patient education, medication adherence remained unaffected. One study found that compliance with patients attending a VGC was in fact better than the FTF clinics [53].

#### Waiting times

Patients waiting times are reduced in VGCs compared to traditional FTF clinics giving higher patient satisfaction [42, 43, 46, 47, 49, 50]. In the Moorfields Eye Hospital’s VGC the patient journey average time was 51 min compared with 92 min in the FTF clinics [46]. At the Singapore National Eye centre, the average journey time was halved to 59 min from 132 min in the VGC as opposed to FTF clinic [43]. The Plymouth Royal Eye Infirmary survey found that 95% of patients felt that waiting times were the same or better than a traditional clinic [50]. In the Edinburgh Princess Alexandra Eye Pavilion survey, 77% of patients agreed that the clinic took less time than usual [49]. These results were also found in our own York Hospital survey, in which 97% of patients were either ‘likely’ or ‘extremely likely’ to recommend the VGC service.

### What type of glaucoma is suitable for VGCs?

Glaucoma risk can be stratified based on a number of factors. There are a several systems we use in clinical practice that categorise risk based on these factors. ‘GLAUC-STRAT-FAST’ is used by The Royal College of Ophthalmologist’s to categorise patients as red (highest risk), amber or green (lowest risk) [54]. High risk patients are patients with advanced glaucoma (>8 dB MD); moderate risk patients are those with a MD of 4–8 dB or <4 dB with angle closure or secondary glaucoma; and, low-risk patients are those with a MD of <4 dB in the context of primary glaucoma, OHT or glaucoma suspects.

Moorfields Eye Hospital Glaucoma Service Guidance and the UK Ophthalmology Alliance Guidance are other examples of risk stratification tools. Based on these systems, decisions can be made about allocation of patients to the VGCs. In general, only patients who are classified as ‘low risk’ are recommended for remote monitoring. This is the recommendation of both the clinical decision tools and the literature [9, 13, 14, 55]. The definition of low-risk patients varies slightly between studies. But, as a general rule it includes patients with OHT, glaucoma suspects, those with early (stable) glaucoma and pseudophakic angle closure patients.

However, there is an additional cohort of patients included in the VGC criteria in the risk stratification tools used in clinical practice. And that is that patients with any grade of glaucoma can be included in the VGC at the discretion of the Consultant [55]. One study included patients with any stage of glaucoma in the VGC provided they had been stable for more than 1 year. They found that over a 31-month observation period there were no safety issues [20].

### The future of glaucoma diagnosis and management

Most patients are willing to use mobile devices as part of management of their glaucoma [56]. A number of devices have been designed to allow patients to monitor glaucoma from the comfort of their own home such as implantable devices [57] and contact lenses to measure IOP efficaciously [58]. Furthermore, visual fields can now be determined using applications on a tablet [59–61] or head mounted devices (such augmented reality (AR) headsets) [62–64]. The results can be fed back to healthcare facilities for real time review by clinicians. There are handheld devices (cameras) or add-on lenses for smart phones to capture retinal images [65]. The binocular simultaneous handheld whole eye OCT scans have the potential to revolutionise remote patient care [66].

Finally, with artificial intelligence (AI), we will be able to establish early diagnosis and predict and detect disease progression sooner. Additionally, this technology can assist the clinicians to improve patients care and provide personalised care [67–71].

## Conclusion

The evidence suggests that VGCs are an efficient, safe and cost-effective way to improve glaucoma services without compromising Health Care Professional or patient satisfaction.

## Data Availability

Data sharing not applicable to this article as no datasets were generated or analysed during the current study.

## References

[1] Steinmetz J, Bourne R, Briant P, Flaxman S, Taylor H, Jonas J (2021). Causes of blindness and vision impairment in 2020 and trends over 30 years, and prevalence of avoidable blindness in relation to VISION 2020: the Right to Sight: an analysis for the Global Burden of Disease Study. Lancet Glob Health.

[2] Partridge L, Deelen J, Slagboom P (2018). Facing up to the global challenges of ageing. Nature.

[3] Buchan J. The way forward. 2017 https://www.rcophth.ac.uk/wp-content/uploads/2021/12/RCOphth-The-Way-Forward-Glaucoma-300117.pdf.

[4] Myint J, Edgar D, Kotecha A, Murdoch I, Lawrenson J (2011). A national survey of diagnostic tests reported by UK community optometrists for the detection of chronic open angle glaucoma. Ophthalmic Physiol Opt.

[5] Gunn P, Marks J, Au L, Waterman H, Spry P, Harper R (2018). Acceptability and use of glaucoma virtual clinics in the UK: a national survey of clinical leads. BMJ Open Ophthalmol.

[6] Keesara S, Jonas A, Schulman K (2020). Covid-19 and health care’s digital revolution. N Engl J Med.

[7] Jones L, Bryan S, Miranda M, Crabb D, Kotecha A (2018). Example of monitoring measurements in a virtual eye clinic using ‘big data. Br J Ophthalmol.

[8] Kotecha A, Bonstein K, Cable R, Cammack J, Clipston J, Foster P (2015). Qualitative investigation of patients’ experience of a glaucoma virtual clinic in a specialist ophthalmic hospital in London, UK. BMJ Open.

[9] Odden J, Khanna C, Choo C, Zhao B, Shah S, Stalboerger G (2020). Telemedicine in long-term care of glaucoma patients. J Telemed Telecare.

[10] Wright H, Diamond J (2015). Service innovation in glaucoma management: using a Web-based electronic patient record to facilitate virtual specialist supervision of a shared care glaucoma programme. Br J Ophthalmol.

[11] Rathod D, Win T, Pickering S, Austin M (2008). Incorporation of a virtual assessment into a care pathway for initial glaucoma management: feasibility study. Clin Exp Ophthalmol.

[12] Kiage D, Kherani I, Gichuhi S, Damji K, Nyenze M (2013). The Muranga Teleophthalmology Study: Comparison of virtual (Teleglaucoma) with in-person clinical assessment to diagnose glaucoma. Middle East Afr J Ophthalmol.

[13] Rathod D, Pickering S, Austin M (2010). Virtual assessment and glaucoma shared care. Eye.

[14] Clarke J, Puertas R, Kotecha A, Foster P, Barton K (2017). Virtual clinics in glaucoma care: face-to-face versus remote decision-making. Br J Ophthalmol.

[15] Gupta S, Sinha S, Dagar A (2013). Evaluation of the effectiveness of diagnostic & management decision by teleophthalmology using indigenous equipment in comparison with in-clinic assessment of patients. Indian J Med Res.

[16] Anton A, Nolivos K, Pazos M, Fatti G, Ayala M, Martinez-Prats E (2021). Diagnostic accuracy and detection rate of glaucoma screening with optic disk photos, optical coherence tomography images, and telemedicine. J Clin Med.

[17] Vinod K, Sidoti P (2022). How glaucoma care changed for the better after the pandemic. Curr Opin Ophthalmol.

[18] Sanayei N, Albrecht M, Martin D, Marin N, Fereshetian S, Baker S (2022). Outcomes of a Hybrid Ophthalmology Telemedicine Model for Outpatient Eye Care During COVID-19. JAMA Netw Open.

[19] Boodhna T, Saunders L, Crabb D (2015). Are rates of vision loss in patients in English glaucoma clinics slowing down over time? Trends from a decade of data. Eye.

[20] Nikita E, Gazzard G, Sim D, Fasolo S, Kortum K, Jayaram H (2023). Expansion of patient eligibility for virtual glaucoma clinics: a long-term strategy to increase the capacity of high-quality glaucoma care. Br J Ophthalmol.

[21] Banes M, Culham L, Bunce C, Xing W, Viswanathan A, Garway-Heath D (2006). Agreement between optometrists and ophthalmologists on clinical management decisions for patients with glaucoma. Br J Ophthalmol.

[22] Spry P, Spencer I, Sparrow J, Peters T, Brookes S, Gray S (1999). The Bristol Shared Care Glaucoma Study: reliability of community optometric and hospital eye service test measures. Br J Ophthalmol.

[23] Kotecha A, Brookes J, Foster P (2017). A technician-delivered ‘virtual clinic’ for triaging low-risk glaucoma referrals. Eye.

[24] Bourne R, French K, Chang L, Borman A, Hingorani M, Newsom W (2010). Can a community optometrist-based referral refinement scheme reduce false-positive glaucoma hospital referrals without compromising quality of care? The community and hospital allied network glaucoma evaluation scheme (CHANGES). Eye.

[25] Devarajan N, Williams G, Hopes M, O’Sullivan D, Jones D (2011). The Carmarthenshire Glaucoma Referral Refinement Scheme, a safe and efficient screening service. Eye.

[26] Henson D, Spencer A, Harper R, Cadman E (2003). Community refinement of glaucoma referrals. Eye.

[27] Trikha S, Macgregor C, Jeffrey M, Kirwan J (2012). The Portsmouth-based glaucoma refinement scheme: a role for virtual clinics in the future?. Eye.

[28] Sakata L, Lavanya R, Friedman D, Aung H, Gao H, Kumar R (2008). Comparison of gonioscopy and anterior segment ocular coherence tomography in detecting angle closure in different quadrants of the anterior chamber angle. Ophthalmology.

[29] Rigi M, Bell N, Lee D, Baker L, Chuang A, Nguyen D (2016). Agreement between gonioscopic examination and swept source fourier domain anterior segment optical coherence tomography imaging. J Ophthalmol.

[30] Porporato N, Baskaran M, Husain R, Aung T (2020). Recent advances in anterior chamber angle imaging. Eye.

[31] Phu J, Wang H, Khou V, Zhang S, Kalloniatis M (2019). Remote grading of the anterior chamber angle using goniophotographs and optical coherence tomography: implications for telemedicine or virtual clinics. Transl Vis Sci Technol.

[32] Founti P, Narayan A, Raja A, Nathwani N, Tur S, Thomas R. Outcomes of newly referred patients with suspected angle closure: do we need to redefine the clinical pathways? Eye. 2020. 10.1038/s41433-023-02713-7. 10.1038/s41433-023-02713-7 PMC10858203 37684375

[33] The Royal College of Ophthalmologists. The management of angle-closure glaucoma. 2020. https://www.rcophth.ac.uk/wp-content/uploads/2021/10/The-Management-of-Angle-Closure-Glaucoma-Clinical-Guidelines.pdf.

[34] He M, Jiang Y, Huang S, Chang D, Munoz B, Aung T (2019). Laser peripheral iridotomy for the prevention of angle closure: a single-centre, randomised controlled trial. Lancet.

[35] Teixeira F, Sousa D, Leal I, Barata A, Neves C, Pinto L (2020). Automated gonioscopy photography for iridocorneal angle grading. Eur J Ophthalmol.

[36] Matsuo M, Mizou S, Nitta K, Takai Y, Sugihara K, Tanito M (2021). Intraobserver and interobserver agreement among anterior chamber angle evaluations using automated 360-degree gonio-photos. PLoS ONE.

[37] Murakami Y, Wang D, Burkemper B, Lin S, Varma R (2016). A population-based assessment of the agreement between grading of goniophotographic images and gonioscopy in the Chinese-American Eye Study (CHES). Investig Ophthalmol Vis Sci.

[38] The Royal College of Ophthalmologists. Standards for virtual clinics in glaucoma care in the NHS Hospital Eye Service. 2016. https://www.rcophth.ac.uk/wp-content/uploads/2021/01/Virtual-Glaucoma-Clinics.pdf.

[39] Thomas S, Jeyaraman M, Hodge W, Hutnik C, Costella J, Malvankar-Mehta M (2014). The effectiveness of teleglaucoma versus in-patient examination for glaucoma screening: a systematic review and meta-analysis. PLoS ONE.

[40] Azzopardi M, Prokosch-Willing V, Michelessi M, Fea AM, Oddone F, Mercieca K. The current use of glaucoma virtual clinics in Europe. Eye. 2023;37:1350–6. 10.1038/s41433-022-02111-5 PMC9188015 35690678

[41] Thomas S, Hodge W, Malvankar-Mehta M (2015). The cost-effectiveness analysis of teleglaucoma screening device. PLoS ONE.

[42] Heimann H, Broadbent D, Cheeseman R (2020). Digital ophthalmology in the UK - diabetic retinopathy screening and virtual glaucoma clinics in the National Health Service. Klin Monbl Augenheilkd.

[43] Huang O, Chew A, Finkelstein E, Wong T, Lamoureux E (2021). Outcomes of an asynchronous virtual glaucoma clinic in monitoring patients at low risk of glaucoma progression in Singapore. Asia Pac J Ophthalmol (Philos).

[44] McKirdy A, Imbuldeniya A (2017). The clinical and cost effectiveness of a virtual fracture clinic service: An interrupted time series analysis and before-and-after comparison. Bone Jt Res.

[45] Owaifeer A, Al-Swailem S, Dehailan A, Naim A, Molhim M, Khandekar R (2022). Physician satisfaction with virtual ophthalmology clinics during the COVID-19 pandemic: a tertiary eye care center experience. Cureus.

[46] Kotecha A, Baldwin A, Brookes J, Foster P (2015). Experiences with developing and implementing a virtual clinic for glaucoma care in an NHS setting. Clin Ophthalmol.

[47] Gunn P, Marks J, Au L, Read S, Waterman H, Spry P (2022). Virtual clinics for glaucoma care - Patients’ and clinicians’ experiences and perceptions: a qualitative evaluation. Eye.

[48] Court J, Austin M (2015). Virtual glaucoma clinics: patient acceptance and quality of patient education compared to standard clinics. Clin Ophthalmol.

[49] Ali A, O’Connell N, Tatham A (2021). Patient satisfaction with virtual compared to face-to-face glaucoma clinics. Acta Ophthalmol.

[50] Spackman W, Waqar S, Booth A (2021). Patient satisfaction with the virtual glaucoma clinic. Eye (Lond).

[51] Rhodes L, Huisingh C, McGwin G, Girkin C, Owsley C (2019). Glaucoma patient knowledge, perceptions, and predispositions for telemedicine. J Glaucoma.

[52] Modjtahedi B, Chu K, Luong T, Hsu C, Mattox C, Lee P, Nakla M (2018). Two-year outcomes of a pilot glaucoma suspect telemedicine monitoring program. Clin Ophthalmol.

[53] Cheng K, Young S, Donaldson S, Malcolm T, Tatham A (2023). Adherence to topical glaucoma therapy in patients attending virtual clinics. Eye.

[54] The Royal College of Ophthalmologists. Designing Glaucoma Care Pathways using GLAUC-STRAT-FAST. 2022. https://www.rcophth.ac.uk/wp-content/uploads/2022/04/Designing-Glaucoma-Care-Pathways-using-GLAUC-STRAT-FAST.pdf

[55] Poostchi A, Kastner A, Konstantakopoulou, Gazzard G, Jayaram H (2023). Clinical risk stratification in glaucoma. Eye.

[56] Dai M, Xu J, Lin J, Wang Z, Huang W, Huang J (2017). Willingness to use mobile health in glaucoma patients. Telemed J E Health.

[57] Enders P, Cursiefen C (2020). Device profile of the EYEMATE-IO™ system for intraocular pressure monitoring: overview of its safety and efficacy. Expert Rev Med Devices.

[58] Mansouri K, Weinreb R, Liu J (2015). Efficacy of a contact lens sensor for monitoring 24-h intraocular pressure related patterns. PLoS ONE.

[59] Prea S, Kong G, Guymer R, Vingrys A (2021). Uptake, persistence, and performance of weekly home monitoring of visual field in a large cohort of patients with glaucoma. Am J Ophthalmol.

[60] Vingrys A, Healey J, Liew S, Saharinen V, Tran M, Wu W (2016). Validation of a tablet as a tangent perimeter. Transl Vis Sci Technol.

[61] Greenfield J, Deiner M, Nguyen A, Wollstein G, Damato B, Backus B (2021). Virtual reality oculokinetic perimetry test reproducibility and relationship to conventional perimetry and OCT. Ophthalmol Sci.

[62] Tsapakis S, Papaconstantinou D, Diagourtas A, Droutsas K, Andreanos K, Moscho M (2017). Visual field examination method using virtual reality glasses compared with the Humphrey perimeter. Clin Ophthalmol.

[63] Alawa K, Nolan R, Han E, Arboleda A, Durkee H, Sayed M (2021). Low-cost, smartphone-based frequency doubling technology visual field testing using a head-mounted display. Br J Ophthalmol.

[64] Tsapakis S, Papaconstantinou D, Diagourtas A, Kandarakis S, Droutsas K, Andreanos K (2018). Home-based visual field test for glaucoma screening comparison with Humphrey perimeter. Clin Ophthalmol.

[65] Haddock L, Kim D, Mukai S (2013). Simple, inexpensive technique for high-quality smartphone fundus photography in human and animal eyes. J Ophthalmol.

[66] Chopra R, Wagner S, Keane P (2021). Optical coherence tomography in the 2020s-outside the eye clinic. Eye.

[67] Campbell C, Ting D, Keane P, Foster P (2020). The potential application of artificial intelligence for diagnosis and management of glaucoma in adults. Br Med Bull.

[68] Ting D, Pasquale L, Peng L, Campbell J, Lee A, Raman R (2019). Artificial intelligence and deep learning in ophthalmology. Br J Ophthalmol.

[69] Liu S, Graham S, Schulz A, Kalloniatis M, Zangerl B, Cai W (2018). A deep learning-based algorithm identifies glaucomatous discs using monoscopic fundus photographs. Ophthalmol Glaucoma.

[70] Martins T, Schor P, Menden L, Fowler S, Silva R (2022). Use of artificial intelligence in ophthalmology: a narrative review. Sao Paulo Med J.

[71] Al-Aswad L, Ramachandran R, Schuman J, Medeiros F, Eydelman M (2022). Artificial intelligence for glaucoma: creating and implementing artificial intelligence for disease detection and progression. Ophthalmol Glaucoma.

